# Factors and barriers that influence the matriculation of underrepresented students in medicine

**DOI:** 10.3389/fpsyg.2023.1141045

**Published:** 2023-05-25

**Authors:** Cynthia Tello, Christine A. Goode

**Affiliations:** ^1^American University of the Caribbean School of Medicine, Cupecoy, Sint Maarten; ^2^Graduate College of Biomedical Sciences and College of Dental Medicine, Western University of Health Sciences, Pomona, CA, United States

**Keywords:** underrepresented, underrepresented in medicine (URiM), diversity, matriculation, professional school, impostor phenomenon, self-efficacy, barriers

## Abstract

Despite many initiatives over more than 4 decades, the diversity of United States physicians still does not reflect the diversity of the United States population. The present study undertakes a literature review of the last 30 years to investigate barriers and protective factors underrepresented college students encounter as applicants for medical school. Known barriers that influence matriculation into medical school were analyzed such as academic metrics and test scores. Additionally, elements that are less well studied were investigated such as factors perceived as barriers by underrepresented applicants in addition to protective factors that allow them to persist in their journey in the face of difficulties and adversity.

## Introduction

It has been known for many years that the United States is facing a significant physician shortage in particular in the primary care field (Zhang et al., 2020; Association of American Medical Colleges, 2021c) and this has been exacerbated by the COVID-19 pandemic (Dill, 2021; Frogner and Dill, 2022). Furthermore, there is a continuing inequity between the diversity of healthcare providers and population trends in the United States. In the 1960s, the importance of these provider inequities was first investigated and the federal government along with organizations such as the Association of American Medical College (AAMC) eventually created initiatives and programming to expose and prepare underrepresented populations for careers in healthcare (Petersdorf, 1992; Nickens et al., 1994; Hayes-Bautista et al., 2000; Smedley et al., 2003; Sullivan, 2011; Bennett et al., 2021). A similar situation is seen in other health professions such as dentistry and pharmacy (Campbell et al., 2021; Nalliah et al., 2021; Bradley-Guidry et al., 2022; Chisholm-Burns et al., 2022). The AAMC describes those underrepresented in medicine (URiM) as “racial and ethnic populations that are underrepresented in the medical profession relative to their numbers in the general population” (Association of American Medical Colleges, 2004). As of 2020, 5.3% of physicians identified as Black or African American, 5.4% identified as Hispanic, Latino, or of Spanish origin, 1.9% identified as Other race or ethnicity, and less than 1% identified as American Indian or Alaska Native or as Native Hawaiian or Other Pacific Islander (0.4 and 0.2%, respectively; Association of American Medical Colleges, 2021a). By comparison, the census bureau reported that Black and African-American citizens accounted for 13.6% of the population and Hispanic/Latino/Latina/Latinx for 18.9% (U.S. Census Bureau, 2021).

There are many reasons that it is important to increase diversity in health professions. For example, studies have shown that URiM graduates are more likely to become primary care providers and serve the underrepresented and underserved communities, often coming from these communities themselves (Komaromy et al., 1996; Xu et al., 1997; Andriole and Jeffe, 2011; Lupton et al., 2012; Kuczewski and Brubaker, 2014; Larson and Frogner, 2019). Additionally, racial concordance and patient compliance has been explored and it has been shown that patients are more likely to comply with their medications and health directives when the race and experiences of their provider matches their own, leading to improved health outcomes (Street et al., 2008; Strumpf, 2011; Alsan et al., 2019; Mindlis et al., 2020; Schoenthaler and Ravenell, 2020; Takeshita et al., 2020). The recent social unrest in the United States, the pandemic which disproportionately affected minorities and calls by various organizations declaring racism a public health threat (American Medical Association, 2020; American Public Health Association, 2021; American Psychiatric Association, 2022) have most recently highlighted the importance of having a socially, ethnically, and culturally diverse healthcare workforce.

Nevertheless, despite a nearly 60% increase in enrollment in US medical schools in the last two decades (Association of American Medical Colleges, 2021b), and a concomitant rise of URiM and low socioeconomic applicants, the representation of these applicants in medical school classes has not kept pace with changes in population distribution (Grumbach et al., 2001; Smedley et al., 2003; Sanchez et al., 2015; Laurencin and Murray, 2017; Pfeffinger et al., 2020; Morris et al., 2021). Of particular concern, the growth of Black and African-American applicants, matriculants, and graduates has significantly lagged behind other groups (Koenig et al., 1998; Rodriguez and Campbell, 2015; Cloutier et al., 2021; Morris et al., 2021; Nakae and Subica, 2021).

There has been considerable discussion of the factors that contribute to the underrepresentation of minorities and marginalized individuals in all of the health fields, which has led to the concept of a “leaky pipeline” from college to medical school with some inclusion of K–12 (Barr et al., 2008; Upshur et al., 2018). Many contributory factors that are barriers to movement through the pipeline at the level of undergraduate education as well as at the admissions level of the professional schools have been identified. These factors include poor academic preparation (including the K-12 experience), lack of exposure to healthcare, lack of training for standardized examinations, admissions committee compositions, racist attitudes, and such. Perhaps the most studied of these is the observed score gaps among members of different racial and ethnic groups and different socioeconomic groups in regard to GPA and on standardized tests including the Graduate Record Examination, Law School Admission Test and the Medical College Admissions Test (MCAT; Koenig et al., 1998; Camara and Schmidt, 1999; Davis et al., 2013). Despite this, MCAT scores and GPA are used by medical schools to determine the readiness of applicants for success in the curriculum as studies continue to show that these metrics are correlated with passing the level 1 licensure exams, the United States Medical Licensing Examination (USMLE), and the Comprehensive Osteopathic Medical Licensing Examination (COMLEX; Casey et al., 2016; Ghaffari-Rafi et al., 2019; Zhong et al., 2021). This emphasis on academic metrics has led to the term “academic redlining” to describe the “systematic exclusion of qualified applicants from underrepresented racial and economic backgrounds” due to the use of MCAT data (Nakae and Subica, 2021). An overhaul of the MCAT in 2015 aimed to remove some of these barriers but, as with the “old” test, URiM students still perform on average below the 50th percentile (Thomas and Dockter, 2019; Association of American Medical Colleges, 2022c) and one study showed that nearly 30% of the applicants who had scores in the middle third of the MCAT (495–504) were URiM (Terregino et al., 2020). A major component of these unchanging scores especially in the CARs section likely relates to issues associated with underfunded and disinvested K-12 schools leading to a well-documented achievement gap for Black and Latinx children (Reardon, 2016; Goode and Landefeld, 2018; Orfield et al., 2019; Jones et al., 2021). On-going long-term assessment of the validity of the MCAT test is aimed at progression through medical school and will thus naturally focus only on matriculated students rather than students who did not get the scores necessary to be admitted.

In an attempt to mitigate the emphasis on these metrics, the AAMC developed a guide for the holistic review of applicants, through which admissions committees use, in addition to metrics, non-academic aspects (Experiences and Attributes) of an application to ensure that candidates from diverse backgrounds and experiences are reviewed (Association of American Medical Colleges, 2016; Grbic et al., 2019; Bates et al., 2020). However, while holistic review is encouraged by the AAMC, there are differences across schools as to the extent to which holistic review practices are implemented (Glazer et al., 2014) and each school still uses their own algorithms to set threshold cutoffs for GPA and MCAT below which an applicant is automatically rejected (Glazer et al., 2014; Goode and Landefeld, 2018). One reason for this is that pressure still exists on admissions committees to matriculate students with a strong likelihood of success (Glazer et al., 2014) since publications such as *U.S. News & World Report* continue to base rankings of medical schools on research funding, school reputation, and student selectivity factors not the number of diverse graduates (Ko et al., 2023). Another is a lack of a common definition and implementation of the practice (Artinian et al., 2017) and this is confounded by a high degree of variation in the institutional climate and declared social mission of US medical schools (Thomas and Dockter, 2019). Holistic review is also under threat by legal challenges to race-conscious admissions (Thomas and Dockter, 2019). Although the use of race as a factor in admissions has been ruled as legal by the Supreme Court [*Regents of the University of California vs. Bakke* (*1978*), *Grutter vs. Bollinger* (2003), *and Fisher vs. University of Texas* (2013)], the fate of existing admissions policies which support student diversity is currently (2023) again under consideration by the US Supreme Court (Curfman, 2022). The effect of a reversal of race-conscious admissions policies can be extrapolated from the observation that in the six states (California, Washington, Florida, Nebraska, Texas, and Michigan) who have state-wide bans of using race in admissions there has been a significant decline in the enrollment of historically minoritized and marginalized students in public medical schools (Steinecke and Terrell, 2008; Garces and Mickey-Pabello, 2015).

A further issue that contributes to continuing inequities in the matriculation of URiM students is the lack of diversity in academic medicine. Data from the AAMC shows that in allopathic medical schools over 83% of the medical school faculty is white or Asian and almost 90% of this same group hold the rank of Professor (Association of American Medical Colleges, 2022a). This lack of URiM faculty could potentially lead to admissions committees that have a different perspective and may demonstrate unconscious racial bias (Corrice, 2009). The lack of faculty diversity also results in a lack of role models, and this is especially an issue with Black males in medicine (Association of American Medical Colleges, 2015). Clearly, with a more diverse faculty, it is likely that the higher level of cultural humility would be a benefit to the educational experience (Odom et al., 2007; Joseph O. R. et al., 2021).

Looking at the financial cost of medical school, in 2019 the average 4-year cost of medical school was $250,222 at a state institution and $330,180 at a private medical school (Youngclaus and Fresne, 2020). Costs like these will disproportionately affect the lower socioeconomic applicants who may already have considerable undergraduate debt. Indeed, looking at the rates of matriculation into medical school as reported by the AAMC (Youngclaus and Roskovensky, 2018), approximately 76–79% of matriculants belonged to the top two highest quintiles in which yearly parental income is greater than $121,000, while only 5% come from the lowest household income quintile. With such clear household income inequities among matriculants, the influence of financial barriers is obvious and will disproportionally affect particularly Black and Hispanic or Latino applicants as a result of the persistent racial wealth gap and the effects of structural racism (Porter et al., 2020).

It is increasingly understood that various social determinants are paramount to the lack of diversity in medicine since social factors have long-term effects. These include several aspects that are unique in certain ways, but all relate to the marginalization of individuals in society, such as stereotype threat, minority status stress, and racial discrimination. Impostor phenomenon (Clance and Imes, 1978) is described as self-doubt of intellect, skills, or accomplishments. Although *this is not a pathological condition,* imposterism does *correlate with burnout*, psychological stress, depression, *and adverse mental health* (Bravata et al., 2020; Cawcutt et al., 2021). An *interesting point of view from some individuals in higher education* (Nadal et al., 2021; Ramos and Wright-Mair, 2021) *suggests that* impostor *phenomenon is a direct byproduct of systemic oppression of marginalized groups*. In addition, the lack of institutional diversity and shortage of role models in higher education and professional school increases the notion of “not belonging” (Stone et al., 2018). *The negative feelings and doubt that come from* impostor *phenomenon especially in the competitive science, technology, engineering*, *and math* (*STEM*) *courses can lead to higher stress levels, anxiety,* depression, *poor attendance, lower course grades*, *and* less persistence in STEM and beyond (Canning et al., 2019a).

In direct contrast to impostor phenomenon, self-efficacy is a key attribute for pre-medical and medical students (Artino, 2012). Self-efficacy as defined by Bandura (1977) is “…an individual’s belief in his or her own ability to organize and implement action to produce the desired achievements and results.” This confidence will be critical for success at all levels of learning and those with higher self-efficacy will be more likely to persist in STEM and onward to medical school (MacPhee et al., 2013). Additionally, social support systems and mentoring are important for students to provide continued motivation to carry on with their pursuit of a healthcare career (McNeill et al., 2014).

Given that many of the initiatives to remove or reduce the barriers to success for URiM students have not made significant changes to the diversity of medical schools, it may be time instead to take a critical look at what can be done to facilitate success by supporting the inherent qualities of URiM applicants. Thus, we aimed to explore the factors underrepresented students face as applicants for medical school and to answer the following research question: What are the factors and barriers that influence the matriculation of URiM students into medical school?

## 2. Methods

### 2.1. Design

Online databases, PubMed, and EBSCO were used to identify papers published in English between 1995 and 2023 regarding matriculation of students to US medical schools. Results were screened using a two-phase system, title and abstract review, with subsequent full text review by both CT and CAG to determine if articles met the inclusion or exclusion criteria.

### 2.2. Search strategy

The search strategy focused on factors that may influence the matriculation of URiM applicants into medical school. Barriers and protective factors to investigate were initially identified by CT through her personal experiences as a first-generation Latina. These were modified and extended by discussions with other URiM pre-medical students and refined as the literature review was performed. The development of categories was an iterative process as the manuscript was prepared and revised.

The initial search took place in October 2020 with a subsequent search performed in March 2023. From December 2022 to April 2023, citation alerts were reviewed for any additional relevant publications. Employing the use of the supplemental Preferred Reporting Items for Systematic Reviews and Meta-Analyses (PRISMA) checklist (Page et al., 2021), a combination of the following search terms and keywords were used: matriculants, underrepresented, resiliency, self-efficacy, medical school, barriers, impostor, African-American, Black, Hispanic, Latino, successful, and health profession school. These search terms were initially determined by the personal and educational experiences of CT and CAG. Limits were set to publications and results confined to those available in English between 1995 and 2023.

### 2.3. Inclusion and exclusion criteria

Both researchers screened the literature with specific inclusion and exclusion criteria, which were modified throughout the study selection process to narrow the focus of the selected articles.

To be included in the review, papers needed to specifically discuss or focus on factors that either supported or reduced the pool of underrepresented applicants to US medical schools. The target participants were URiM high school and college students. Articles discussing strategies for retention of matriculants in medical school or strategies for increasing enrollment in other health professions were not included. Additionally, papers were excluded if they discussed factors that did not match one of the theme areas we had identified, were not published in English, or pertained to schools other than those in the United States.

Screening took place in two phases. Phase 1 consisted of screening title and abstract of articles retrieved during the search of keywords. The screening was for specific terminology related to URiM admissibility and/or matriculation into medical school in the United States. In Phase II, full-text articles were assessed for eligibility and relevance to the goals of the study and research question.

## 3. Results

### 3.1. Included studies

Initial search results were exported to EndNote and duplicates removed for a total of 196 citations. Upon further exploration and browsing of appropriate foundation websites, citation alerts, and review of cited references, 30 additional publications were added for a total of 226 citations. Phase I screening excluded 78 articles and 148 were deemed appropriate for full review by both authors. After Phase II, full text review confirmed that 66 articles met all criteria. A flow diagram was adapted from the PRISMA guidelines (Supplementary Figure 1 in Supplementary material) and used during the screening process. Several of the studies discussed multiple factors. Articles included presentations, scholarly perspectives, qualitative surveys, and data reports. Most of the studies were single institution, and many included a control group or a comparison to national data. The literature was grouped into broad categories by CT and CAG based on the barriers or protective factors discussed. Tables 1–5 summarize each article reviewed by describing the type, and size of study and the goal, and the results.

**Table 1:** Academic metrics (12 articles; 18.2% total).

First author, Year of publication^1^	Type	Summary
Agahi et al. (2018)	Single institution study of 206 graduating medical school students.	Undergraduate GPA, undergraduate science GPA, and MCAT scores had small to moderate association with medical school grades and performance on COMLEX-USA Level 1.
	Goal: To determine correlation of undergraduate GPA and MCAT scores with academic and clinical performance in medical school.	
Busche et al. (2020)	Multisite study: examined national data from a single MCAT examination of 7,970 matriculants. (24.5% URiM). Summative performance compared 596 non-URiM vs. 261 URiM matriculants in 16 medical schools.	National sample showed 98% of non-URiM students compared to 94% of URiM students showed on time progression to year 2.
		The percentage of students who progressed to year 2 on time was 93% or above for those students with MCAT total scores from 494 to 528.
	Multisite study: examined national data from a single MCAT examination of 7,970 matriculants. (24.5% URiM). Summative performance compared 596 non-URiM vs. 261 URiM matriculants in 16 medical schools.	Students with a wide range of MCAT scores progressed to year 2 on time.
		Correlations of MCAT scores with summative performance in year 1 ranged from medium to large.
Capers and Way (2015)	Single institution study of two groups of African-American (AA) students: HBCU graduates (N = 39) versus graduates from Predominantly White Institutions (PWI) (N = 173).	Despite lower average MCAT scores, the AA HBCU graduates had similar rates of graduation, residency matching and achieving board certification, as their PWI cohort.
	Goal: To compare the academic and postgraduate performance of HBCU graduates versus PWI graduates.	
Giordani et al. (2001)	Single institution study of 458 first-year medical students who had matriculated either traditionally (443 students) or after completing postbaccalaureate (PB) work (15 URiM students).	Undergraduate grade-point average ± SD:
	Goal: To measure the effectiveness of a formal post-baccalaureate (PB) experience for URiM students before medical school.	PB matriculants 2.9 ± 0.2 vs. traditional matriculants 3.6 ± 0.4.
		Comparisons revealed that the traditional students (control group) scored significantly higher on MCAT than did the PB students.
Girotti et al. (2015)	Single institution study of 274 medical students (81.4% URiM).	In comparison with control students, CA students entered primary care specialties significantly more often (50.4% versus 38.3%)
	Goal: To evaluate a ‘Conditional Admissions’ (CA) program.	70% of the CA students passed the UMLE step 1 first time and 74% passed the UMLE step 2 first time.
	Control Group: Students (2,953) not admitted through CA.	
Girotti et al. (2020)	Multisite study: Examined national data from 2017 MCAT examinations of 80,767 test-takers (22.3% URiM).	Large differences in average total MCAT scores were observed when comparing test takers from higher-resource schools (502.1 average) to those from lower-resource schools (492.3 average). Lower-resource schools had the least selective admissions practices and had campuses that were primarily nonresidential.
	Goal: To compare test takers from higher-resource undergraduate schools (95.6% total) to those from lower-resource schools (4.4% total).	The largest score gaps were between test takers who identified as white (503.3 average) and URiM test takers: black or African American (494.2 average) and Hispanic, Latino, or Spanish (496.7 average).
Goode and Talbot (2016)	Single institution study of 36 graduates (61% URiM) from a special master’s program (SMP) who matriculated to medical school.	28 of the 36 graduates completed their medical training in 4 years and placed into residencies. Five students decelerated and one withdrew.
	Goal: To measure the effectiveness of a SMP program.	Sixteen graduates (57.1%) went into a primary-care field: family medicine, internal medicine, and pediatrics.
		The SMP graduates averaged in the 25–50th percentile rank in the medical school class pre-clinical curriculum.
Grumbach and Chen (2006)	Multi-institution study: Comparison of 265 students (67% URiM) in a formal postbaccalaureate (PB) program.	Cumulative undergraduate GPAs of the two groups were not significantly different.
	Goal: To measure the effectiveness of a formal post-baccalaureate (PB) experience before medical school.	Initial MCAT scores available for 237 PB participants (21.9 ± 4.33) and 295 controls (22.1 ± 5.81) showed a slight difference between the two groups.
	Control group: 396 non PB participants.	Within 3 years, three times as many PB program participants as controls had matriculated into medical school (67.6 vs. 22.5%) with a mean MCAT of 24.0 ± 4.32 SD.
Kadavakollu et al. (2022)	Single institution study of 78 diverse students (over 40% URiM, 95% from MUA) in a premedical enrichment program.	MCAT scores were self-reported after the program by 38 (50.7%) students. Of these, the average MCAT score was 504 ± 6.2. 3 students (7.9%) scored below the 34th percentile, 24 (63.2%) scored between the 35th and 68th percentile, and 11 (28.9%) scored above the 68th percentile, with three of those students scoring above the 90th percentile.
	Goal: To evaluate the effectiveness of the enrichment program by looking at MCAT scores and professional school matriculation.	Medical school matriculation was self-reported after the enrichment program by 27 (36.0%) students. Twenty-five (92.6%) of those matriculated into medical school, one student to a Doctor of Podiatric Medicine program, and one to a Doctor of Naturopathic Medicine.
Schneid et al. (2022a)	Single institution study of 75 (31% URiM) students in a premedical enrichment program.	MCAT scores Prematriculation students: 31.9 ± 3.3 vs. Control Group: group 34.5 ± 3.1
	Goal: To measure the effectiveness of a prematriculation program by looking at MCAT scores and performance in Year 1 of medical school.	Participants performed significantly better than control group in Year 1 courses that were covered in the prematriculation program compared to courses that were not covered.
	Control Group: group 293 non-participants.	The overall performance in the prematriculation program correlated significantly with Year 1 performance and was found to be a strong predictor for Year 1 performance.
Schneid et al. (2022b)	Multi-site study of 25 postbaccalaureate (PB) students from program associated with the medical school (32% URiM), and 34 students who participated in other PB programs (47% URiM).	MCAT scores PB students from medical school associated program: mean 85.0 (54–99) vs. Control Group: group 91.7 (51–100).
	Goal: To measure the effectiveness of a PB programs by looking at MCAT scores, pre-clerkship course performance and United States Medical Licensing Exam (USMLE) Step 1 results.	The exam performance in pre-clerkship courses of the PB students from the medical school program was not significantly different from the control. Students who participated in other PB programs showed a lower performance compared to the rest of the class.
	Control Group: 329 non-PB participants (16% URiM).	Both groups of PB program participants scored significantly lower on USMLE Step 1 exam compared to control. However, no significant differences were found regarding USMLE Step 1 passing rates between the three groups.
Upshur et al. (2018)	Single institution study of 53 undergraduate students (81.1% URiM) in a pipeline program for academic enrichment.	80% of 25 pipeline students graduated within 6 years of enrollment. The degree completion rate was almost double the campus average of 42% and more than double that of the non-program URiM students (37%).
	Goal: To measure the effectiveness of a pipeline program by looking at undergraduate degree completion.	All remaining participants were on target for timely graduation, which would yield a 91% on-time graduation rate.
	Control Group: Non-program students (URiM and non-URiM).	

^1^Publications referenced by first author and year.

**Table 2:** Faculty diversity (eight articles; 12.1% total).

First author, Year of publication^2^	Type	Summary
Acheampong et al. (2019)	Single institution study of 16 Black male medical school graduates.	Participants noted that institutional expectations were lower for historically minoritized and marginalized medical students and that predominately white medical schools lacked appropriate support mechanisms for nurturing the success of Black men.
	Qualitative study.	
	Goal: To determine the factors that contributed to and methods for coping with stress by black males in medical school.	
Agrawal et al. (2005)	National data from a survey of Student Affairs Deans from 86 Allopathic and Osteopathic medical schools.	Barriers were reported as low MCAT scores (90% of schools), absence of role models (77% of schools) and not enough historically minoritized faculty members (71% of schools).
	Qualitative study.	The average URiM of the responding schools was 10.4% which was similar to 10.9% at all schools (HBCUs and those located in Puerto Rico were excluded).
	Goal: To investigate barriers to URiM student recruitment.	
Bauer et al. (2019)	Multi-site study of 506 high school students.	Primary perceived barrier to college success and healthcare career training by respondents was financial issues, with some eliminating options early because financial concerns were so high.
	Qualitative survey	
	Goal: To investigate perceived barriers to rural students pursuing health related careers.	
Dickins et al. (2013)	Single-institution study of 18 medical school graduates (100% URiM).	Twelve of the respondents (67%) commented on the importance of student body diversity.
	Qualitative survey.	Sixteen of the 18 interviewees (88%) strongly articulated the importance of the collaborative learning climate.
	Goal: To identify potential strategies through which medical schools can support the success of URM medical students.	Six of the participants (33%), endorsed a healthcare disparities curriculum.
		Nine of the 18 respondents (50%) spoke about the need to increase historically minoritized faculty.
Freeman et al. (2016)	Cross-Sectional, multi-institutional study of 82 pre-medical undergraduate students (100% URiM).	Respondents noted lack of access to advising mentors and health related opportunities as significant barrier. Family conflict and cost were also noted.
	Qualitative survey.	
	Goal: To identify potential barriers for undergraduate students to applying to professional school.	
Jacobs et al. (2022)	Single institution study of 179 search committee members.	Committee members attended and evaluated a mandatory 2-h workshop on best practices in search processes and implicit bias training.
	Goal: To evaluate the influence of diversity training of search committee members on increasing URM applicants interviewed and hiring of URM faculty.	A significant increase in the diversity of applicants, candidates interviewed, and faculty hired, was observed 3 years following implementation of the search committee workshop.
Ko et al. (2023)	Multi-site study of 39 deans and directors of admissions from 37 medical schools.	Barriers identified were pressure from faculty and administrators to overemphasize academic scores, lack of leadership commitment, and political and social influences, such as donors and alumni.
	Qualitative study.	
	Goal: To describe processes and structures that hinder efforts to increase diversity in admissions.	
Roche et al. (2021a)	Cross-Sectional study of 52 (100% URiM) graduating high school seniors.	Primary perceived barrier to healthcare career training by respondents was competitiveness and financial cost.
	Qualitative study.	
	Goal: To investigate perceived barriers to URiM high school students pursuing health related careers.	

^2^Publications referenced by first author and year.

**Table 3:** Educational (11 articles; 16.7% total).

First author, Year of publication^3^	Type	Results
Andriole et al. (2015)	National data from the Association of American Medical Colleges (AAMC) Pre-MCAT Questionnaire of 213,497 respondents who subsequently completed the MCAT.	16.2% (34,539) of the respondents were URiM and just over a third of these (11,842) participated in research in college.
	Goal: To evaluate the effect of participation in college laboratory research apprenticeship.	There was a positive, independent association between research participation and medical-school acceptance, independent of their MCAT scores. However, the magnitude of the observed relationship was modest (a 12% higher likelihood of acceptance).
Chan et al. (2022)	Single institution study of 184 medical students who graduated from either high quality or low-quality high schools as determined by high school reading proficiency (HSRP >50% is considered high quality school).	Statistically significant differences in average exam performance between the high-quality and low-quality school-attended groups were found for the MCAT (508.24 vs. 509.86, *p* = 0.025).
	Goal: To determine if there are group differences in MCAT scores between high-and low-quality high schools.	
Cosentino et al. (2015)	Mixed-methods external evaluation of the impact of the RWJF Summer Medical and Dental Education Program (SMDEP). The outcomes analysis was for 6,826 undergraduate students (64% URiM) who participated in SMDEP.	Eighty-three percent of participants earned a bachelor’s degree compared to national data of about 59 percent of all students (40 percent for blacks and American Indian or Alaskan Native and 52 percent for Hispanics).
		Fifty-five percent of participants applied to either medical or dental school or both. Of those who apply, 68% subsequently matriculated.
Freeman et al., 2016	Cross-Sectional study of 82 (100% URiM) undergraduate students.	Respondents noted lack of access to advising mentors and health related opportunities as significant barrier. Family conflict and cost were also noted.
	Qualitative survey.	
	Goal: To identify potential barriers for undergraduate students to applying to medical school.	
Goode and Landefeld (2018)	Commentary	Discussion of the lack of diversity in healthcare. Noted that educational barriers for URiM students include implicit bias, disparities in reading levels (k-12), need for remedial English and math courses in college, and ineffective advising along the pipeline.
Joseph J. et al. (2021)	Single-institution study of 35 undergraduate or postbaccalaureate participants (48% URiM).	Participants noted lack of diversity in the medical fields.
	Qualitative survey.	Lack of complete understanding and information needed to navigate the application process to become a medical professional.
	Goal: To determine barriers/facilitators to pursuing a medical career.	Participants indicated access to mentoring and guidance by medical trainees and professionals as likely to facilitate a medical career.
Kadavakollu et al., 2022	Single institution study of 78 students in a premedical enrichment program for diverse students (Over 40% URiM, 95% from medically underserved areas, MUAs).	38 of the 78 students self-reported MCAT scores for an average of 504 ± 6.2. Three students (7.9% of total) scored below the 34th percentile, 24 (63.2%) scored between the 35 and 68th percentile, and 11 (28.9%) scored above the 68th percentile, with three of those students scoring above the 90th percentile.
	Goal: To evaluate the success of an enrichment program aimed at students from rural or MUAs.	Medical school matriculation was self-reported after the enrichment program by 27 (36.0%) students. Sixteen of those 27 (59.2%) matriculated into osteopathic medical schools, nine (33.3%) into allopathic medical schools, one student to a Doctor of Podiatric Medicine program, and one to a Doctor of Naturopathic Medicine.
	Comparison group: national MCAT averages.	
Mason et al. (2022)	Multi-site study: Examined national data for 211,216 students (24.8% identified as first-generation college graduates) who took the MCAT and completed the AAMC Pre-Medical College Admissions Test Questionnaire.	Students with (vs. without) paid work experience outside hospitals/labs/clinics were less likely to apply, be accepted, and matriculate into medical school.
	Goal: To evaluate the role of paid work or summer programs on likelihood of medical school application.	Students who participated in a college research apprenticeship, a summer academic-enrichment program, and paid or volunteer work in hospital/clinic/lab settings were more likely to apply to medical school.
Schneid et al. (2022a)	Single institution study of 75 academically disadvantaged students (31% URiM) in a prematriculation enrichment program	Prematriculation students MCAT average 31.9 ± 3.3 vs. Control: group 34.5 ± 3.1.
	Goal: To evaluate the effectiveness of a summer prematriculation program for academically disadvantaged students by looking at MCAT scores and performance in Year 1 of medical school.	Prematriculation participants performed significantly better than control group in Year 1 courses that were covered in the prematriculation program compared to courses that were not covered.
	Control group: 293 non-participants.	The overall performance in the prematriculation program correlated significantly with Year 1 performance and was found to be a strong predictor for Year 1 performance.
Thurmond and Cregler (1996)	Single institution study of 55 diverse high-school students (100% URiM) in a premedical research apprentice program.	All 55 participants matriculated to a college or university. Twenty-nine (53%) of these students graduated from college. Seventeen of 29 students (59%) matriculated into medical school.
	Goal: To determine effect of a research experience in the student pipeline.	30.1% of white students graduate from high school, and 14.6% obtain a baccalaureate degree compared with 28% of blacks who graduate from high school, and 7.5% who graduate from college.
	Comparison group: State-wide high-school and graduation rates.	
Toretsky et al. (2018)	Literature review and interviews to summarize known barriers to URiM students entering the health professions.	In California, the barriers include: (1) Lack of academic preparation; admissions requirements, especially for doctoral degree programs; (2) Lack of concordant mentors; (3) Stereotype threat; (4) Limited exposure to health careers; and (5) Poor advising.

^3^Publications referenced by first author and year.

**Table 4:** Financial (12 articles; 18.2% total).

First author, Year of publication^4^	Type	Results
Baugh and Baugh (2022)	Scholarly opinion	Authors conclude that the current financial aid system’s reliance on high debt burden undermines goals to recruit and matriculate URiM students to medical school.
Carnevale and Strohl (2013)	Report of analysis of enrollment trends at 4,400 postsecondary institutions by race and institutional selectivity.	Key findings:
		82% of new white enrollments have gone to the 468 most selective colleges, while enrollments for Hispanics (72%) and African Americans (68%) have gone to 2-year and 4-year open-access schools.
		Selective colleges spend anywhere from two to almost five times as much on instruction per student as the open-access colleges.
		More than 30% of AA and Hispanics with a high school GPA higher than 3.5 go to community colleges compared to 22% of whites with the same GPA.
Carnevale and Smith (2018)	Policy brief	Low-income students are less likely to complete college and are more susceptible to dropping out.
		Low-income students are less likely to enroll in 4-year or selective institutions or to graduate with a bachelor’s degree.
		Low-income students who work while enrolled are more likely to work outside of their fields.
		Working while enrolled generally tends to benefit higher-income students and tends to harm or disadvantage low-income students, but low-income students often have less of a real choice about whether and how much to work.
Fenton et al. (2016)	Single institution study of 14,919 medical school applicants.	A method of adjusting applicant GPA and MCAT scores for SES based on application data was simulated and tested.
	Goal: To determine if an applicants’ cumulative pre-medical grade point average and total MCAT score could be adjusted for socioeconomically status (SES).	The adjustment methods reduced or eliminated disparities in URiM and disadvantaged student representation.
Grbic and Roskovensky (2012)	National Study of 14,389 first-time applicants who were not accepted to medical school.	5,282 (37%) of the 14,389 first-time nonaccepted applicants became repeat applicants and 40% (2,121) of these were accepted to medical school.
	Goal: To explore factors associated with becoming a repeat applicant to medical school.	Applicants who had more than $20,000 of educational debt were less likely to become repeat applicants.
Grbic et al. (2015)	National data of 38,558 medical school applicants for whom a socioeconomic indicator could be assigned.	Compared with Asian and white applicants, African American and Hispanic applicants were more often categorized as low EO status.
	Goal: To validate a socioeconomic indicator based on parental education (E) and occupation (O) for use in medical school admissions	There were moderate to strong associations between the EO categories and indicators of socioeconomic disadvantage.
Joseph O. R. et al. (2021)	Single-institution study of 35 undergraduate or postbaccalaureate participants (48% URiM).	Lack of appropriate financial resources for the application processes including admissions testing, traveling for interviews, medical school fees, and lodging expenses were cited as major barriers.
	Qualitative survey.	
	Goal: To determine barriers/facilitators to pursuing a medical career.	
Michalec and Hafferty (2023)	Mixed-methods study (quantitative and qualitative approaches).	Authors conclude that the PMP is constructed to favor those from high socioeconomic status, privileged backgrounds, and those majoring in typical premed majors such as in the Biological Sciences.
	Goal: To identify potential (explicit and implicit) exclusionary practices, processes, and mechanisms in the premedical pathway (PMP).	
Poll-Hunter et al. (2023)	Scholarly perspective from the action collaborative for black men in medicine (launched by AAMC and the National Medical Association).	Multiple hurdles exist including financial considerations, academic hurdles and information access.
	Goal: To address the systems and factors that influence the trajectory to medicine for Black men.	
Talamantes et al. (2014)	National study of 40,491 matriculant and applicant files and 17,518 matriculating students.	Among Latino matriculants to medical school, 65.6% (1,028/1,566) did not attend a CC.
	Goal: To assess associations between student characteristics and participation in a more economically viable community college (CC) pathway.	Applicants who attended a CC had a significantly longer number of years in college before application to medical school 4.6 ± 1.8 (*N* = 12,598) vs. 6.8 ± 3.9 (*N* = 1,920).
		Compared with the 27% of white matriculants who used CC pathways, 34% of Latinos and 28% of Black matriculants used CC pathways.
Thomas and Dockter (2019)	Commentary	Authors recommend that medical schools must maintain or increase support for STEM academic enrichment programs at all levels. Additionally, they should support a holistic admissions process that considers race and socioeconomic status.
Toretsky et al. (2018)	Literature review and interviews to summarize known barriers to URiM students entering the health professions.	Financial challenges are even more pronounced for URiMs than other racial/ethnic groups as they are more likely to have lower socio-economic status.

^4^Publications referenced by first author and year.

**Table 5:** Psycosocial factors (23 articles; 34.8% total).

First author, year of publication^5^	Type	Summary
Abdulrazzak et al. (2021)	Single institution study of 49 high school students (64% URiM) participating in a mini-medical school program.	The most significant barriers identified were cost, family education, and lack of guidance and role models.
	Qualitative study.	Mid-and post-program studies indicated a significant reduction in the self-reported barriers related to guidance and mentorship. One-on-one mentoring provided to the high school students a means of support and may have empowered them to recognize their own potential.
	Goal: To identify barriers to pursuing a medical career among URiM high school students.	
Acheampong et al. (2019)	Single institution study of 16 Black male medical school graduates.	Race was a major stressor as evidenced by academic expectations that differed, social isolation and institutional atmosphere, less access to academic resources as well as academic inequity and racial tension.
	Qualitative study.	The participants discussed the negative impact of racialized stress on their academic performance as well as physical and emotional health.
	Goal: To determine the factors that contributed to and methods for coping with stress by black males in medical school.	Coping mechanisms included social and spiritual support.
Afghani et al. (2013)	Single institution study of 253 high school students (22% URiM) in a summer enrichment program. 36 college students (59% URiM) and 12 medical students (92% URiM) participated as near-peers and coaches.	The coaches reported that their self-confidence and leadership increased, they became more aware of the importance of cultural diversity and their motivation for a career in medicine increased.
	Qualitative study.	
	Goals: To assess the effect of involvement in a pipeline program for high-school students on attitudes of peer coaches.	
Artino (2012)	Scholarly perspective on self-efficacy.	A discussion of the nature and structure of self-efficacy and several instructional implications for medical education. These include using peer modeling to build self-efficacy and using social persuasion to help students believe that they can cope with difficult situations.
Bernard et al. (2018)	Single institution study of 157 Black undergraduates at a predominately White university.	Racial discrimination was positively related to higher subsequent levels of IP, controlling for initial levels of IP.
	Qualitative survey.	
	Goal: To longitudinally examine the relationship between racial discrimination and Impostor Phenomenon (IP).	
Bright et al. (2018)	Scholarly perspective on social support mechanisms.	A discussion of effective programs and strategies used to increase admissions of underrepresented students in general, and black males in particular, into medical school.
Canning et al. (2019b)	Single-institution study of 61 STEM faculty and 15,466 students (10.9% URiM) enrolled in all the courses taught by the STEM faculty respondents.	Course evaluations revealed that URiM students were demotivated and had more negative experiences in classes taught by fixed (versus growth) mindset faculty.
	Qualitative survey.	The racial achievement gap in courses taught by the fixed mindset faculty was twice as large as the achievement gaps in courses taught by more growth mindset faculty.
	Goal: To test if STEM faculties’ fixed beliefs about intelligence and ability would lead to stereotype threat and URiM students would experience lower motivation and underperform relative to their non-stereotyped peers.	
Cokley et al. (2013)	Single-institution study of 50 Black and 76 Hispanic/Latina/Latino/Latinx undergraduate participants.	Black students reported significantly more race-related stress than Hispanic/Latina/Latino/Latinx students. There were no differences in impostor feelings between the two groups.
	Qualitative survey.	Impostor feelings were significantly positively correlated with minority status stress and negatively correlated with psychological wellbeing.
	Goal: To examine to what extent minority status stress and impostor syndrome was predictive of the students’ mental health.	Impostor feelings predicted mental health problems more strongly than stress related to one’s racial/ethnic status.
Cokley et al. (2017)	Single-institution study of 106 Black and 108 Hispanic/Latina/Latino/Latinx undergraduate participants.	Black students reported higher perceived discrimination than Hispanic/Latina/Latino/Latinx students. There were no differences in impostor feelings by racial/ethnic group. Among the two groups impostor feelings were not predictive of depression but were for anxiety.
	Qualitative survey.	
	Goal: To determine the extent of impostor syndrome and perceived discrimination among the racial/ethnic minoritized groups. To determine if impostor feelings moderated the relationship between perceived discrimination and depression and anxiety.	
Cole and Griffin (2013)	Scholarly literature review on student-faculty interactions.	Investigates the experiences of URiM individuals and notes how the frequency, quality, and outcomes of student-faculty interactions vary based on the social identities of those interacting. Discusses the importance of the students-faculty interaction in particular for URiM students.
Echegoyen et al. (2019)	Single institution study of self-efficacy in 1,652 freshman participants (84–96% URiM) in a first-year research intensive program.	Participants in the research program showed a statistically significant increase in self-efficacy and a decrease in IP.
	Goals: To measure self-efficacy prior to and at the conclusion of the program.	Participants demonstrated increased long-term retention relative to non-participants (84.8% compared to 58.5% at year 3).
	To measure retention in STEM.	
	Control group for retention was non-program participants.	
Freeman et al., 2016	Cross-Sectional, multi-institutional study of 82 pre-medical undergraduate students (100% URiM).	Respondents noted lack of access to advising mentors and health related opportunities as significant barriers. These challenges led to the students questioning the viability of medicine as a career.
	Qualitative survey.	
	Goal: To identify importance of peer-mentoring for undergraduate students applying to professional school.	
Kosobuski et al. (2017)	Single institution study of 22 undergraduate students (72.7% URiM) in a pre-matriculation program.	Program participants showed retention of microbiology content and increased confidence about the overall medical school experience after participating in the program.
	Goal: To measure the effectiveness of a pre-matriculation program in improving self-efficacy by looking at performance in a basic science class.	
Lige et al. (2017)	Multi-institution study of 112 Black undergraduate students in both public and private predominantly White institutions (PWIs).	Ethnic-racial identity was positively and significantly associated with self-esteem and inversely associated with IP. Self-esteem was negatively and significantly associated with IP.
	Qualitative survey.	
	Goal: To examine the relationships between racial identity, self-esteem, and impostor phenomenon (IP) among Black undergraduate students.	
McClain et al. (2016)	Single institution study of 218 Black college students.	Minority stress and IP were significantly negatively related to mental health whereas racial/ethnic identity were positively related to mental health.
	Qualitative survey.	
	Goal: To examine racial/ethnic identity, minority status stress, and impostor phenomenon (IP) as predictors of mental health.	
Morgan et al. (2016)	Scholarly perspective	The authors describe the premedical experience for URiM students and note the positive effects of developing mentor relationships with faculty and students from professional schools.
Morgan Consoli et al. (2015)	Single institution study of 121 undergraduates (100% URiM).	Hope—intrinsic determination to meet one’s goals and planning of multiple methods in which to reach these goals-was a significant predictor for both resilience and thriving.
	Qualitative study.	
	Goal: To investigate the role of several factors (spirituality, hope, social support, and cultural values) in predicting resilience and thriving.	Spirituality and cultural pride were significant predictors only for thriving.
Mushonga and Henneberger (2020)	Multi-institution study of 156 traditional college students and 79 non-traditional college students (ages 26+; 100% URiM) attending historically Black colleges and universities (HBCUs) and predominantly White institutions.	Results indicate spirituality, social support, self-esteem, and racial identity are positive factors associated with positive mental health in Black students.
	Qualitative study.	No significant differences were found in mental health among Black students attending historically Black colleges and universities and predominantly White institutions.
	Goal: To identify protective mechanisms promoting positive mental health.	The non-traditional students reported higher rates of moderate mental health than the traditional students.
		There was no difference in mental health between students attending HBCUs than those in predominantly White institutions.
Peteet et al. (2015a)	Single institution study of 112 Black undergraduate students.	The results reveal that higher IP scores predicts higher psychological distress, and lower self-esteem.
	Qualitative survey.	
	Goal: To examine the extent to which Impostor Phenomenon (IP) predicts psychological distress and self-esteem.	
Peteet et al. (2015b)	Single institution study of 161 URiM undergraduate students. The majority of participants (68%) were college juniors/seniors and had a GPA ≥ 3.0	The results revealed that low psychological well-being and low ethnic identity are predictors of IP.
	Qualitative survey.	
	Goal: To examine the extent to which measures of first-generation status, psychological well-being, and ethnic identity predict Impostor Phenomenon (IP) scores among high-achieving URiM undergraduates.	
Roche et al. (2020)	Single institution study of pipeline program for 10 high school seniors (100% URiM) from families with no parent having completed college.	Participants showed statistically significant changes in two major components of self-efficacy—“Belief in Personal Ability” and “Belief that Ability Grows with Effort.”
	Qualitative study.	Pre-program assessment showed that the students displayed high levels of self-efficacy from the outset suggesting that students who successfully applied and matriculated into the program already had high levels of self-efficacy.
	Goal: To determine what effect participation in a pipeline program has on the levels of self-efficacy in URiM high-school students.	
Thomas and Dockter (2019)	Multi-site study of 329 Latinx middle school students (100% URiM).	Ethnic-racial identity related to higher levels of academic self-efficacy.
	Qualitative study.	
	Goal: To investigate links between ethnic-racial identity and academic self-efficacy.	
Yelorda et al. (2021)	Single-institution mixed methods study of 31 high school students (100% URiM) in a STEM enhancement summer program.	Most students scored in the high self-efficacy group for at least one domain:
	Goal: To measure self-efficacy across three domains.	65% for academic self-efficacy (defined as an individual’s belief that they can successfully perform academic tasks at specific levels).
		56% for social self-efficacy (defined as an individual’s confidence in his/her ability to engage in the social interactional task necessary to initiate and maintain interpersonal relationships).
		The lowest group (19%) was for emotional self-efficacy (defined as an individual’s convictions in one’s emotional functioning capabilities).
		Participants noted several themes necessary for educational success: fulfillment in academic challenges, focus on future goals, learning from failures, and asking for help from teachers and peers (social support).

^5^Publications referenced by first author and year.

#### 3.1.1. Academic metrics

A significant and much studied barrier for increasing diversity is academic metrics since medical schools rely heavily on MCAT scores and undergraduate GPA to determine admission, but data continue to show a persistent score gap for URiM and low SES applicants (Table 1). There is some evidence that standardized test scores and GPA do not necessarily correlate with progression through medical school. Indeed, students with midrange and lower MCAT scores also proceed successfully through their pre-clinical curriculum and pass their first licensure exams (USMLE and COMLEX; Capers and Way, 2015; Girotti et al., 2015, 2020; Agahi et al., 2018; Busche et al., 2020; Schneid et al., 2022a,b). A reduced emphasis on MCAT would capture more URiM students; however, the key role of the medical school must be recognized in supporting students with lower scores—a collaborative learning environment, diverse peers, and an experiential curriculum have all been highlighted as critical. The demonstrated success of diversity-targeted pipeline and postbaccalaureate programs in preparing students for degree completion and success in medical school additionally points to the critical importance of improving study-methods and test-taking skills (Giordani et al., 2001; Grumbach and Chen, 2006; Goode and Talbot, 2016; Upshur et al., 2018; Kadavakollu et al., 2022; Schneid et al., 2022b).

#### 3.1.2. Faculty diversity

One issue, stemming from the previously mentioned lack of faculty diversity in academic medicine, is that admissions committees may have different perspectives of the barriers that URiM students will face in acceptance to, and success in, medical school (Table 2). To demonstrate this, when surveyed, admissions leaders viewed academic performance as measured by undergraduate GPA and MCAT as the most significant measure of academic attainment and hence a key barrier for URiM applicants (Agrawal et al., 2005; Ko et al., 2023). In contrast, diverse students when asked the same question were more likely to identify financial concerns, feelings of academic inadequacy, and lack of racially concordant faculty and mentors (Dickins et al., 2013; Freeman et al., 2016; Acheampong et al., 2019; Bauer et al., 2019; Roche et al., 2021a). Improving faculty diversity in academic medicine is obviously critical but in the short-term admissions committee members may benefit from unconscious bias mitigation training and workshops including taking implicit association tests (Jacobs et al., 2022). Although the long-term effects of this training are not clear, especially with the frequent turn-over of admissions committees’ members, increasing individuals’ knowledge of their implicit racial biases would likely positively impact their review of historically minoritized and marginalized applicants.

#### 3.1.3. Educational

Many URiM applicants come from lower socioeconomic backgrounds and attend underfunded and disinvested schools and institutions, significantly limiting the number of resources available to these students which can result in lower rates of college attendance, undergraduate degree completion, and lower MCAT scores (Freeman et al., 2016; Goode and Landefeld, 2018; Chan et al., 2022; Table 3). The lack of informed academic advisors and less familiarity with the university and medical school application processes is also a significant barrier for URiM students to overcome, particularly those that are first-generation (Joseph J. et al., 2021).

Underrepresented in medicine applicants are also less likely to have access to positive educational opportunities such as research experience, exposure to healthcare career opportunities or have a family member who is a healthcare provider (Freeman et al., 2016; Goode and Landefeld, 2018; Toretsky et al., 2018). An early association was noted by Thurmond showing that participants in an URiM student research apprenticeship program were more likely to choose a science major and later choose a healthcare career (Thurmond and Cregler, 1996). This has been further reinforced by additional studies demonstrating that URiM students who obtained health professions exposure, and participated in healthcare career opportunities and other summer enrichment programs, had higher MCAT scores and were more likely to apply to medical school (Andriole et al., 2015; Cosentino et al., 2015; Kadavakollu et al., 2022; Mason et al., 2022; Schneid et al., 2022a).

#### 3.1.4. Financial

In addition to the obvious concerns of the cost of medical school (Baugh and Baugh, 2022), financial barriers are evident as URiM students are more likely to come from low socioeconomic backgrounds, experience financial stress during their undergraduate education and are more likely to need to work through college (Carnevale and Smith, 2018; Toretsky et al., 2018; Table 4). This leads to a myriad of results: reduced study time resulting in a less than competitive GPA, no opportunity for extra-curricular and volunteer activities, and the inability to pay for MCAT preparatory classes or even the exam itself. Indeed, the pre-medical pathway has been referred to as an example of discriminatory design based on the disadvantages it poses for applicants who come from low socioeconomic status (SES) backgrounds (Michalec and Hafferty, 2023). Unsuccessful first-time applicants to medical school with high pre-existing debt are further shown to be less likely to reapply (Grbic and Roskovensky, 2012). The additional cost of other programs such as postbaccalaureate courses to increase preparation, meet prerequisites or raise GPA becomes an added financial stressor (Talamantes et al., 2014; Toretsky et al., 2018; Joseph J. et al., 2021; Poll-Hunter et al., 2023). Even pipeline programs that are meant to increase matriculation of URiM students to health profession schools, come at a cost, directly or indirectly through loss of summer wages, and are likely to increase debt. There has been some discussion that low SES is actually the primary barrier to diversifying schools and that it should be considered in admissions instead of race (Carnevale and Strohl, 2013; Grbic et al., 2015; Fenton et al., 2016). However, this would not account for systemic marginalization of URiM applicants related to issues such as education, employment and criminal justice. Indeed, it has been reported that an admissions process that considers SES in place of race does not admit the same numbers of Black and Hispanic/Latina/Latino/Latinx applicants as a process that considers race (Thomas and Dockter, 2019).

#### 3.1.5. Psychosocial factors

In this category, which covers the highest percentage of the citations, we looked at psychosocial factors which are less studied but are clearly significant for historically minoritized and marginalized students (Table 5). We have included the negative factor of impostor phenomenon and the more positive facilitators of self-efficacy and social/spiritual support.

Underrepresented and first-generation students often experience feeling like impostors in college and this can negatively influence their academic performance, their confidence in their abilities, and ultimately their success. The feeling of additional pressure to prove others wrong and the experience of microaggressions and perceived racism is particularly prevalent in minoritized and marginalized communities (Peteet et al., 2015b; Toretsky et al., 2018; Acheampong et al., 2019). An interesting point of view from some individuals in higher education (Ramos and Wright-Mair, 2021) suggests that impostor phenomenon is a direct byproduct of systemic oppression (e.g., racism, sexism) and is exacerbated by bias and microaggressions (Bernard et al., 2018). As an example, in courses taught by STEM faculty with a belief in fixed ability, the racial achievement gap was large, the URiM students experienced stereotype threat, were demotivated and this contributed to the feelings of impostorism (Canning et al., 2019b). Indeed, it is clear that impostor phenomenon, minority status stress, and race related stressors such as racial microaggressions are interconnected and all contribute to poor mental health, psychological stress, and low self-efficacy (Cokley et al., 2013, 2017; Peteet et al., 2015a; McClain et al., 2016; Bernard et al., 2018). Of importance though is that racial identity, the importance of race/ethnicity to an individual’s overall sense of self, may mitigate the feelings of impostor phenomenon (McClain et al., 2016) by increasing self-efficacy (Echegoyen et al., 2019), and self-esteem (McClain et al., 2016; Lige et al., 2017). In contrast, lower racial/ethnic identity is a predictor of impostor phenomenon (Peteet et al., 2015b).

Self-efficacy as defined by Bandura (1977) is “an individual’s belief in his or her own ability to organize and implement action to produce the desired achievements and results.” The literature suggests that although many URiM students may need additional assistance for successful matriculation, those that apply to and participate in pipeline programs or research experiences possess high levels of confidence in their ability to succeed and their participation in these programs reinforces this quality of self-efficacy (Artino, 2012; Turan et al., 2013; Miller, 2014; Kosobuski et al., 2017; Echegoyen et al., 2019; Roche et al., 2020; Yelorda et al., 2021). As mentioned, racial identity is also related to higher self-efficacy and targeted pipeline programs may provide an environment to foster and improve this ethnic centrality. Indeed, Bright et al. (2018) have suggested the development of programs to specifically target these positive qualities by increasing the opportunities for academic nurturing and mentoring to increase application and matriculation of URiM applicants.

To provide continued motivation to carry on with their pursuit of medical school, URiM and marginalized applicants rely on social support systems and mentoring (Afghani et al., 2013; Acheampong et al., 2019; Abdulrazzak et al., 2021; Roche et al., 2021b). For some students, in the face of adversity and stress, connecting with others decreased the negative effects and helped them stay motivated (Morgan Consoli et al., 2015; Acheampong et al., 2019). Relating to other students with experience in similar paths, perhaps through peer-mentoring programs, helps to mitigate negative experiences associated with being a marginalized pre-medical student perhaps by increasing or reinforcing racial/ethnic identity. With regard to medical school applications, as URiM applicants look at the grades needed and statistics for acceptance, the impostor phenomenon may lead them to believe that they are not competitive and this may be enough to deter them from moving forward in their endeavor if they are not actively supported at multiple points in their journey (Freeman et al., 2016; Morgan et al., 2016). The student-faculty relationship in particular has been shown to be one of the most influential and important social relationships for students, especially for students of color (Cole and Griffin, 2013). In this regard, undergraduate research experiences can provide long-lasting mentor-student relationships. Additionally, for some URiM students, having faith and/or practicing spirituality seems to decrease stress associated with the pre-medical journey and provides the support to successfully continue moving forward in academics (Acheampong et al., 2019; Mushonga and Henneberger, 2020).

## 4. Discussion

The lack of physician diversity in the US has been investigated extensively and the vast majority of literature is aimed at investigating barriers that negatively affect URiM students matriculating to and graduating from medical school. This review, which focused on the application and matriculation of URiM applicants to medical school, reinforced the known barriers of academic criteria, educational disadvantages, lack of faculty diversity, and financial considerations. For a low SES applicant, which encompasses many URiM applicants, healthcare experience opportunities, involvement in research, summer enrichment programs, and even co-curricular activities are often out of reach due to the need to work to support themselves and their families (Clement, 2016). Lack of these opportunities may make an applicant’s file less competitive especially in light of the AAMC experiences-attributes-metrics model (Association of American Medical Colleges, 2020). An alternative may be encouraging admissions officers and committee members to recognize the inherent value of paid healthcare experience as compared to medical volunteerism and clinical shadowing (Association of American Medical Colleges, 2022b) especially given the observation that these experiences may improve success in the clerkship environment (Strowd et al., 2020). A commitment of all levels of administration in medical schools to full implementation of holistic admissions practices will certainly allow for a better evaluation of the various aspects of an applicant’s files including the barriers that they have faced. Diversifying the admissions committee membership will also be key (Robinett et al., 2021) as well as recognizing and mitigating unconscious bias at all levels. The emphasis on objective academic criteria such as GPA and standardized testing should be tempered by the realization that these metrics are the end result of a number of interrelated factors such as school district funding, study time, educational support and financial ability to participate in opportunities such as advanced placement courses and test-preparation (Allegretto et al., 2022). More investigation must be done to delineate these relationships and address the issues at the source. In addition, these metrics do not provide information on the quality of the healthcare professional an applicant may become, and non-cognitive variables are critical in addressing this component of the application (Strowd et al., 2020). A promising new initiative, the AAMC PREview professional readiness exam (Association of American Medical Colleges, 2023), aims to objectively assess certain key core competencies and skills for pre-medical students such as resilience, service orientation, ethics teamwork, and cultural competence. The efficacy of this exam is currently being tested in 18 allopathic schools.

In addition to the obvious barriers, there are also a number of psychological, cultural, and social factors that contribute to the premedicine journey of URiM students. Impostor phenomenon is increasingly reported in historically minoritized and marginalized communities and is considered by some as a consequence of structural racism (McGee et al., 2021) and lack of campus and professional diversity. In the absence of role models, historically minoritized and marginalized students may feel like they do not belong, and this can lead to feelings of inadequacy, incompetency, and self-doubt—all criteria of impostor phenomenon. In the STEM educational pipeline where there is a clear lack of racial diversity these feelings of impostor syndrome can and do lead to negative experiences, lack of motivation, burn-out, and ultimately a lack of persistence and/or retention (Goode and Landefeld, 2018; Tao and Gloria, 2019). In contrast, positive racial (and STEM) identity is a protective factor against impostor phenomenon and again highlights the need to increase diversity and racially concordant mentors and role-models at all points in the pipeline, including through residency and into academic medicine.

Other facilitators of URiM students to continue their journey to medical school include self-efficacy and/or self-belief, which is developed in many pipeline programs and research experiences. Clearly to support the cultural wealth of historically minoritized and marginalized students, the involvement of strong role models through high-school and college and social support networks which can foster this development must be nurtured and encouraged. In addition, spirituality and high levels of racial or ethnic centrality have been seen to be important in giving historically marginalized students the tools to deal with perceived and real racism. Finally, any attempts to increase the matriculation of URiM students into medical school must be coupled with a commitment from the institutions that they will provide the climate that will support the persistence and success of those students.

This review was not meant to be exhaustive but instead focused on several factors that we identified that effect underrepresented students on the premedicine pathway. Limitations of this review were that much of the literature discussed came from qualitative reports and from self-reported data and surveys, information which is subjective. In many cases, the cohorts under study were small and were from a single institution or single program. The primary focus was on the racial and ethnic minorities of Black/African American and Hispanic/Latinx, since although Native Americans and Alaskan Natives are also underrepresented in medicine, we were unable to find sufficient literature to speak on additional factors related to this population. The expectation is that the same factors will apply to these groups. In addition, in some regions, Southeast Asians are considered URiM but data reports and most literature aggregate Asian populations. We also did not include other historically minoritized and marginalized groups such as sex assigned at birth/gender/gender identity (LGBTQ+) or disabled. Although diversity initiatives extend back over four decades, this review focused on the last 30 years to cover only two iterations of the MCAT. Finally, our grouping of articles was largely subjective and based on the broad categories, and the articles themselves may not have used the same terms and/or language when discussing the various barriers or protective factors.

To conclude, if the diversity of US physicians is ever to approach the diversity of the US population, all factors should be investigated to increase the academic success and eventual matriculation of historically minoritized and marginalized students to medical school. The emphasis on barriers to success should be balanced with promoting protective factors, in particular, those that diverse students rely on for persistence in their education.

## Author contributions

CT and CG contributed to conception and design of the study. CT proposed the factors and barriers based largely on her experiences as a first-generation Latina and wrote the first draft of the manuscript. CG organized the database. All authors contributed to the article and approved the submitted version.

## Conflict of interest

The authors declare that the research was conducted in the absence of any commercial or financial relationships that could be construed as a potential conflict of interest.

## Publisher’s note

All claims expressed in this article are solely those of the authors and do not necessarily represent those of their affiliated organizations, or those of the publisher, the editors and the reviewers. Any product that may be evaluated in this article, or claim that may be made by its manufacturer, is not guaranteed or endorsed by the publisher.
