# Supplementary material for: Factors and barriers that influence the matriculation of underrepresented students in medicine
Source: Front Psychol. 2023 May 25;14:1141045. doi: 10.3389/fpsyg.2023.1141045 (PMC10247986; doi:10.3389/fpsyg.2023.1141045)

Figure 1 Adapted PRISMA (Preferred Reporting Items for Systematic Review and Meta-Analyses) flow diagram for literature review exploring barriers and protective factors that influence URiM applicant matriculation into medical school. Additional records were also identified through citation alerts and citation references. All numbers reported in the figure include articles from database searches and additional citations.

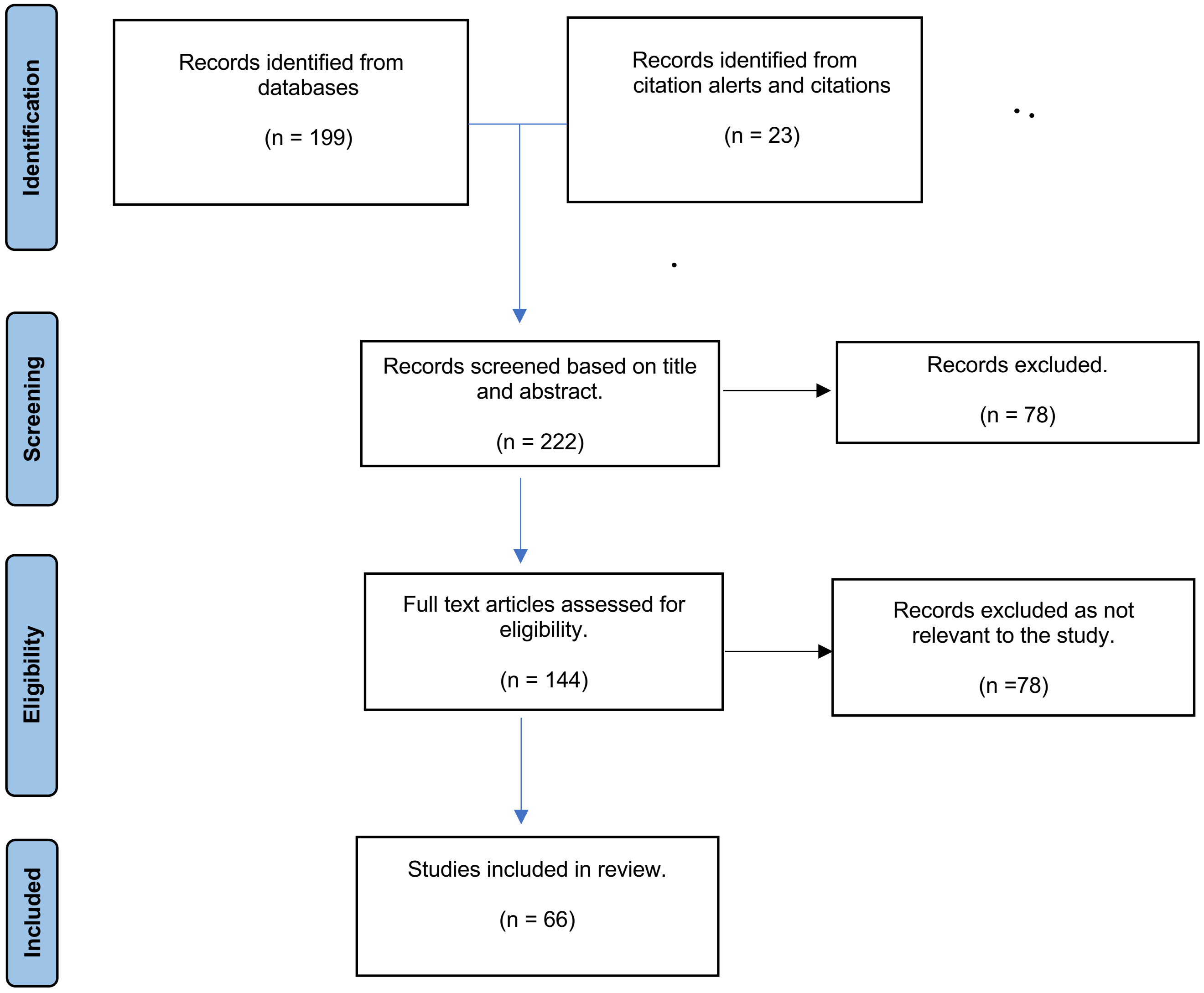

Supplement: Supplementary file 1 [file Image_1.PDF]
